# Human upper-airway respiratory airflow: *In vivo* comparison of computational fluid dynamics simulations and hyperpolarized ^129^Xe phase contrast MRI velocimetry

**DOI:** 10.1371/journal.pone.0256460

**Published:** 2021-08-19

**Authors:** Qiwei Xiao, Neil J. Stewart, Matthew M. Willmering, Chamindu C. Gunatilaka, Robert P. Thomen, Andreas Schuh, Guruprasad Krishnamoorthy, Hui Wang, Raouf S. Amin, Charles L. Dumoulin, Jason C. Woods, Alister J. Bates

**Affiliations:** 1 Division of Pulmonary Medicine, Center for Pulmonary Imaging Research, Cincinnati Children’s Hospital, Cincinnati, OH, United States of America; 2 Department of Infection, Immunity & Cardiovascular Disease, POLARIS Group, Imaging Sciences, University of Sheffield, Sheffield, United Kingdom; 3 Pulmonary Imaging Research Laboratory, University of Missouri School of Medicine, Columbia, Missouri, United States of America; 4 Department of Computing, Imperial College London, London, United Kingdom; 5 MR Clinical Science, Philips, Rochester, MN, United States of America; 6 MR Clinical Science, Philips, Cincinnati, OH, United States of America; 7 Department of Pediatrics, University of Cincinnati School of Medicine, Cincinnati, OH, United States of America; 8 Department of Radiology, Cincinnati Children’s Hospital, Cincinnati, OH, United States of America; 9 Department of Radiology, University of Cincinnati College of Medicine, Cincinnati, OH, United States of America; Technion Israel Institute of Technology, ISRAEL

## Abstract

Computational fluid dynamics (CFD) simulations of respiratory airflow have the potential to change the clinical assessment of regional airway function in health and disease, in pulmonary medicine and otolaryngology. For example, in diseases where multiple sites of airway obstruction occur, such as obstructive sleep apnea (OSA), CFD simulations can identify which sites of obstruction contribute most to airway resistance and may therefore be candidate sites for airway surgery. The main barrier to clinical uptake of respiratory CFD to date has been the difficulty in validating CFD results against a clinical gold standard. Invasive instrumentation of the upper airway to measure respiratory airflow velocity or pressure can disrupt the airflow and alter the subject’s natural breathing patterns. Therefore, in this study, we instead propose phase contrast (PC) velocimetry magnetic resonance imaging (MRI) of inhaled hyperpolarized ^129^Xe gas as a non-invasive reference to which airflow velocities calculated via CFD can be compared. To that end, we performed subject-specific CFD simulations in airway models derived from ^1^H MRI, and using respiratory flowrate measurements acquired synchronously with MRI. Airflow velocity vectors calculated by CFD simulations were then qualitatively and quantitatively compared to velocity maps derived from PC velocimetry MRI of inhaled hyperpolarized ^129^Xe gas. The results show both techniques produce similar spatial distributions of high velocity regions in the anterior-posterior and foot-head directions, indicating good qualitative agreement. Statistically significant correlations and low Bland-Altman bias between the local velocity values produced by the two techniques indicates quantitative agreement. This preliminary *in vivo* comparison of respiratory airway CFD and PC MRI of hyperpolarized ^129^Xe gas demonstrates the feasibility of PC MRI as a technique to validate respiratory CFD and forms the basis for further comprehensive validation studies. This study is therefore a first step in the pathway towards clinical adoption of respiratory CFD.

## Introduction

Many airway diseases result in obstruction of the large airways, including obstructive sleep apnea (OSA), medialized vocal folds, tracheomalacia, laryngomalacia, bronchomalacia, and subglottic stenosis [1–3]. These diseases often result in multiple sites of obstruction, and/or may occur with comorbid lung abnormalities. Currently, there are no clinical methods to assess the contribution of each site of obstruction to respiratory symptoms. Therefore, in cases with multiple levels of obstruction, surgical treatment cannot be directed to the site of obstruction that is most likely to have the most significant impact upon respiratory symptoms [3–6]. In cases with comorbid airway and lung abnormalities, it is often not clear which abnormality is responsible for respiratory symptoms.

Current clinical gold-standard methods of airway evaluation such as spirometry are limited to global assessments of the entire airway, and provide little information on the level of the airway that causes symptoms. Other methods such as endoscopic evaluation, are invasive and qualitative [7]. In vivo regional measurements are rare due to the difficulty in instrumenting the airway without disrupting its natural physiology and airflow.

Computational fluid dynamics (CFD) simulations of respiratory airflow have the potential to solve this unmet medical need and in turn, revolutionize the regional assessment of airway function and response to insult, disease, and treatment in pulmonary medicine and otolaryngology [8–12]. CFD simulations allow regional evaluation of the relationship between anatomy and airflow by mapping airflow parameters such as airway resistance, pressure loss, work of breathing, and wall shear stress [13–17]. Mapping these parameters over the central airways allows identification of regions of high resistance to be identified, quantification of breathing effort through each part of the airway, and can predict the outcome of surgical interventions to address these obstructions [11,18–20].

Respiratory CFD simulations produce quantitative results based on boundary conditions which can be obtained non-invasively; e.g. via medical imaging and external respiratory airflow measurements [21–23]. CFD has been adopted clinically for the assessment of hemodynamics [24–27] and is approved for use by the US Food and Drug Administration (FDA) [28]; however, adoption in respiratory medicine has been limited to date by difficulty in validating the results.

*In vivo* validation of respiratory CFD simulations is difficult due to the challenges in directly measuring airflow within the large airways as stated above; instruments placed inside the airway to measure airflow disrupt natural physiology and airflow. Most comparisons between CFD simulations and physical airflow measurements have instead been performed *in vitro*, often yielding good agreement between the methods [29,30]. However, the limitation of *in vitro* studies is that experimental setups often capture only a subset of physiology, incorporating anatomical or idealized airway shape and size, but neglecting airway motion, heating and humidification. Therefore, a method to validate CFD simulations *in vivo* is necessary to take into account the natural respiratory physiology.

Phase contrast magnetic resonance imaging (PC MRI) offers a non-invasive means to validate CFD simulations by measuring the velocity of a moving gas or liquid comprising MR-detectable (spin-$\frac{1}{2}$) nuclei [31–33]. ^1^H PC MRI is routinely used to assess blood flow for cardiovascular [34,35] or neurological [36] applications. However, the density of MR-detectable nuclei in air is insufficient for imaging and thus the airway lumina do not exhibit signal on conventional MRI. To overcome this barrier, hyperpolarized noble gas nuclei–most commonly ^3^He and ^129^Xe, wherein the nuclear spin polarization is increased by several orders of magnitude–can be used as inhaled contrast agents for airway PC MRI [37–39]. PC MRI of inhaled hyperpolarized gases provides a direct measurement of gas flow *in vivo* which can be compared to CFD simulation results to quantify systematic differences. The feasibility of comparison between airway CFD and hyperpolarized ^3^He gas-and water- based PC MRI measurements has been demonstrated *in vitro* using realistic airway models derived from imaging data [40–43]. In addition, preliminary comparisons of hyperpolarized ^3^He PC MRI and CFD have been reported *in vivo* in rats [39], but to the best of our knowledge, comparison of CFD and *in vivo* hyperpolarized gas PC MRI in humans has yet to be reported.

In this study, we compared the respiratory airflow velocity fields computed by CFD simulations and measured using *in vivo* PC MRI of inhaled ^129^Xe in three human subjects. The velocity fields were compared both qualitatively, in terms of similarity of flow patterns and localization of high-velocity regions, and quantitatively, to assess the correlation and the systematic differences between the techniques. The goal of this study was to assess the feasibility of using PC MRI of inhaled hyperpolarized gases as a method to validate respiratory CFD simulations *in vivo* in humans.

## Materials and methods

To compare CFD simulation results and PC MRI velocimetry, CFD simulations were created with boundary conditions (i.e., airway anatomy and airflow rates) that matched the subjects’ anatomy and physiology as they underwent PC MRI velocimetry. To that end, subject anatomies were also obtained from proton MRI in the same imaging session as ^129^Xe PC MRI velocimetry. In addition, subject respiratory flow rates were recorded during PC MRI velocimetry. Details of PC MRI, CFD, and methods to ensure CFD boundary conditions represent the conditions of the PC MRI velocimetry are given below.

The region of interest for this study ranged from the nasopharynx to the proximal trachea. This choice was based on both clinical and practical considerations. This region of anatomy is typically the region in which airway collapse is observed in subjects with OSA, and includes the soft palate, the retroglossal region, and the epiglottis. Therefore, in the future, we anticipate that validated CFD simulations of this region could provide clinically useful assessments of which of the above regions are contributing to respiratory symptoms. Practically, the descending upper airway provides a large enough lumen to allow large voxel sizes yielding sufficient MRI signal to create images. Conversely, the nasal airways comprise small, tortuous passages that that are difficult to capture within an imaging plane, and the distal trachea and bronchi are subject to respiratory motion that may result in image blurring. Furthermore, the region of interest includes sites of airway narrowing such as the vocal folds, which were expected to result in complex high-velocity regions, and broader regions which were expected to produce low local velocities. Therefore, this choice of geometry should yield a complex and heterogeneous velocity field, allowing a robust comparison between methods. The sagittal plane was chosen for PC MRI acquisitions, as this best captures regions with differing expected velocities within a single imaging plane; CFD was performed in 3D and a sagittal plane was extracted for comparison to PC MRI as discussed below.

### Subjects

Three healthy subjects (male; aged 25, 30 and 35) and with no respiratory abnormalities or smoking history, were recruited. This study was conducted with Cincinnati Children’s Hospital investigational review board approval and with written informed consent obtained by local clinical research coordinators.

### Breathing maneuver and flow rate measurement

**Fig 1:**
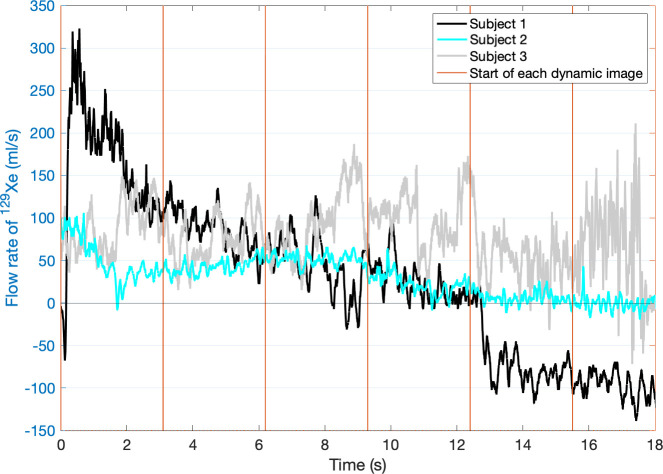
Flow profile measurement and synchronization with PC MRI. Flow rate of inhaled ^129^Xe in each of the three subjects beginning at the time the first PC MRI acquisition started. The vertical red lines represent the start of each subsequent dynamic PC MRI image acquisition.

Each subject wore an anesthesia mask during imaging. The purpose of the mask was to record the respiratory flow rate during inhalation of hyperpolarized ^129^Xe, for use as a CFD boundary condition. To this end, the mask was connected to an MRI-compatible pneumotachograph (Respiratory Flow Head 300L, ADInstruments, Dunedin, New Zealand) which recorded the respiratory flow rate using LabChart software (ADInstruments) [44]. To synchronize flow rate measurements with the MRI acquisition, the MRI scanner outputs a 5-volt trigger signal at the start of each dynamic image acquisition. The LabChart software recorded the flow rate data and these trigger signals simultaneously (Fig 1). Synchronizing flow rate measurements and image acquisition allowed temporal alignment of CFD simulation results and PC MRI velocimetry.

The distal end of the pneumotachograph was connected to a Tedlar® bag containing hyperpolarized ^129^Xe during imaging, and open to room air in-between scans. For each scan, a 1L bag of hyperpolarized ^129^Xe was supplied. Each subject was asked to inhale the 1L of ^129^Xe via their nose as slowly as possible to give a long period (10–20 s) for imaging.

### Phase contrast MRI

**Fig 2:**
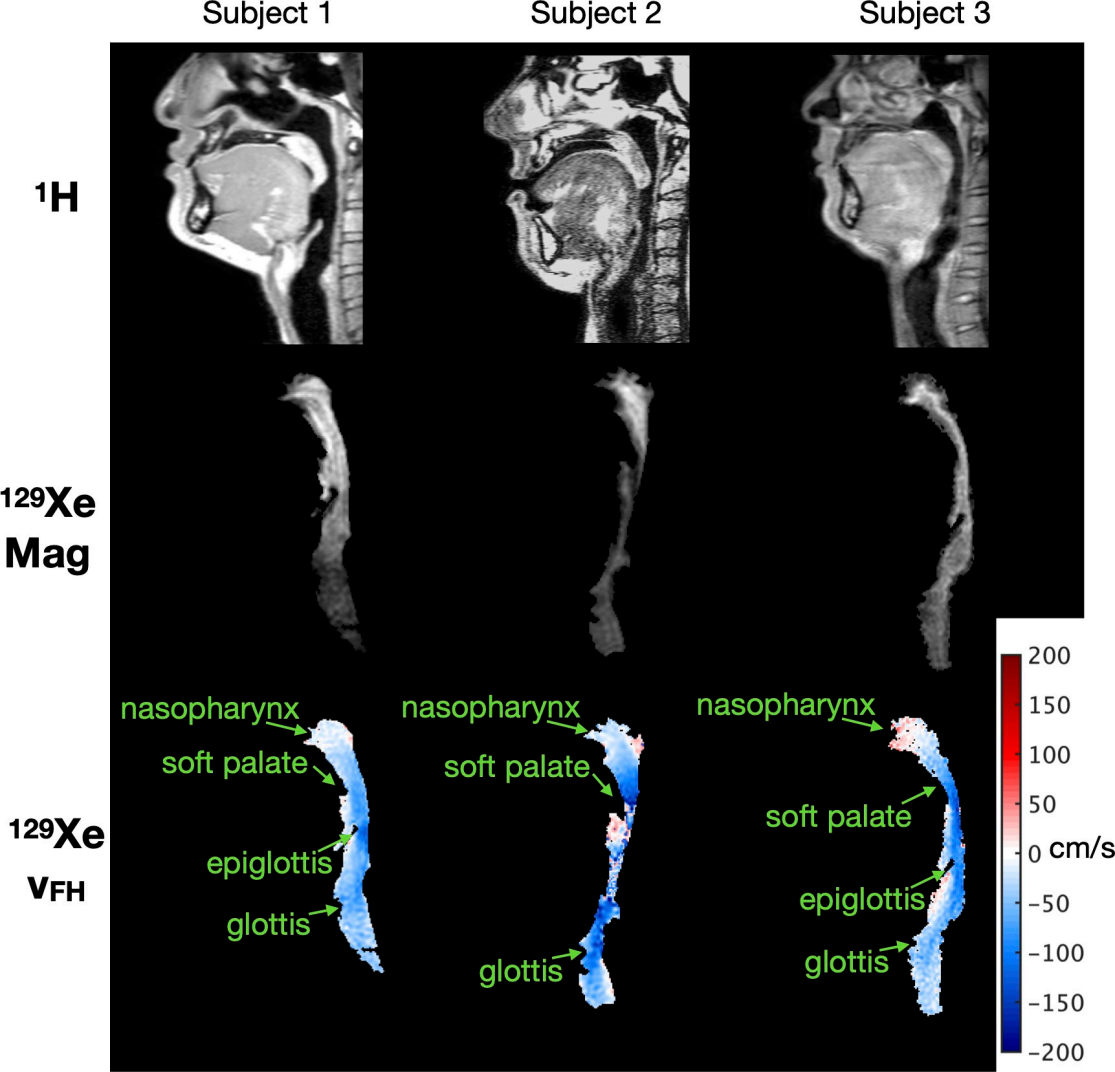
Example of MRI data acquired from each of the three subjects. Top row: Sagittal ^1^H MRI of the head and neck anatomy including the upper airway, captured to create the virtual airway surface for CFD simulations. Middle row: Sagittal magnitude (“Mag”) MR images of inhaled ^129^Xe in the upper airway at a selected dynamic time (note: slices do not perfectly correspond to ^1^H MRI slices above). Bottom row: Foot-Head velocity (v_FH_) maps corresponding to the magnitude images above (one dynamic image), reconstructed from ^129^Xe gas PC MRI data. Red velocities represent flow in the foot to head direction, blue represents flow in the head to foot direction.

PC MRI velocimetry of hyperpolarized ^129^Xe was performed on a 3T MRI scanner (Philips, Best, The Netherlands) using a flexible ^129^Xe radiofrequency coil (Clinical MR Solutions, LLC, Brookfield, WI) positioned around the neck and head. A 1L bag of hyperpolarized ^129^Xe gas–polarized to ~30% using a Polarean 9820 polarizer (Polarean Imaging Ltd, Durham, NC.)–was inhaled over a period of 10–20 seconds a described above, while a time series of PC MR images was acquired. Using a spiral readout combined with Hadamard-interleaved velocity encoding (aliasing velocity 200 cm/s) in up to three spatial directions [45,46], 2D PC MR images were acquired in the sagittal plane. Slices were positioned to cover the extent of the upper airways from the top of the nose to below the epiglottis in the superior-inferior direction. An in-plane resolution of 1.25 or 1.5 mm^2^ and a slice thickness of 10 or 12.5 mm were used. The slice thickness and in-plane resolution were chosen as a compromise between the desire for fine voxels for comparison with the high-resolution CFD simulations, and the need to maintain sufficient signal-to-noise (SNR) for data quantification. For reference, we note that it is common practice to use an in-plane resolution ~3–4 mm and slice thickness of 10–15 mm for ^129^Xe MR imaging of lung ventilation. A time-series of up to 10 images of each slice was acquired; depending on scan parameters, the acquisition time for a single slice with three-directional velocity encoding ranged from 3–5 seconds. Fig 2 shows example ^1^H anatomical MRI images, along with representative dynamic ^129^Xe gas PC MR images, in each of the three subjects.

### Creating virtual airway anatomy via proton MRI

To image the airway anatomy, which is necessary to generate anatomic boundary conditions for the CFD simulations, high-resolution proton MRI data were captured in each subject in the same scanning session as PC MRI. A proton density volume isotropic turbo spin echo acquisition (PD-VISTA) sequence was used with a resolution of 0.35×0.35×0.8 mm. Images were acquired using a head and neck vascular coil that was not compatible with the ^129^Xe coil, and therefore, additional, lower resolution proton images were acquired with the ^129^Xe coil *in situ* using the scanner body coil, to help register ^129^Xe and high-resolution proton images.

To create subject-specific virtual airway surfaces for use in CFD simulations, the high-resolution proton MR images were segmented using a user-guided active contour technique [47] in ITK-Snap 3.6.0 (Penn Image Computing and Science Laboratory, USA, www.itksnap.org), yielding a high-resolution airway surface extending from the external nose and mouth to the upper trachea. A virtual model of the anesthesia mask used for gas delivery was then attached to the exterior of the face to match the experimental setup.

### CFD simulation–boundary conditions, convergence, and turbulence modeling

Boundary conditions for all CFD simulations were the no-slip condition at the airway and mask wall. All surfaces were considered rigid. The mask opening was used as the inlet boundary, which was held at atmospheric pressure. The time-varying flow rate was applied at the outlet in the upper trachea.

All simulations were transient simulations covering the duration of the ^129^Xe inhalation. The airway was filled with air at the start of the inhalation and all flow through the inlet was comprised of ^129^Xe gas, which allowed the mixing of ^129^Xe and the air already in the airway to be incorporated in the model. The mixing of the gases was modeled using the multi-component gas module within Simcenter Star-CCM+ 14.06 (Siemens PLM Software, Plano, TX, USA) [48]. Diffusion coefficients were calculated using Chapman-Enskog theory and ranged from 0.06 cm^2^/s for self-diffusion of xenon to 0.14 cm^2^/s for xenon in infinite dilution in air [49].

**Fig 3:**
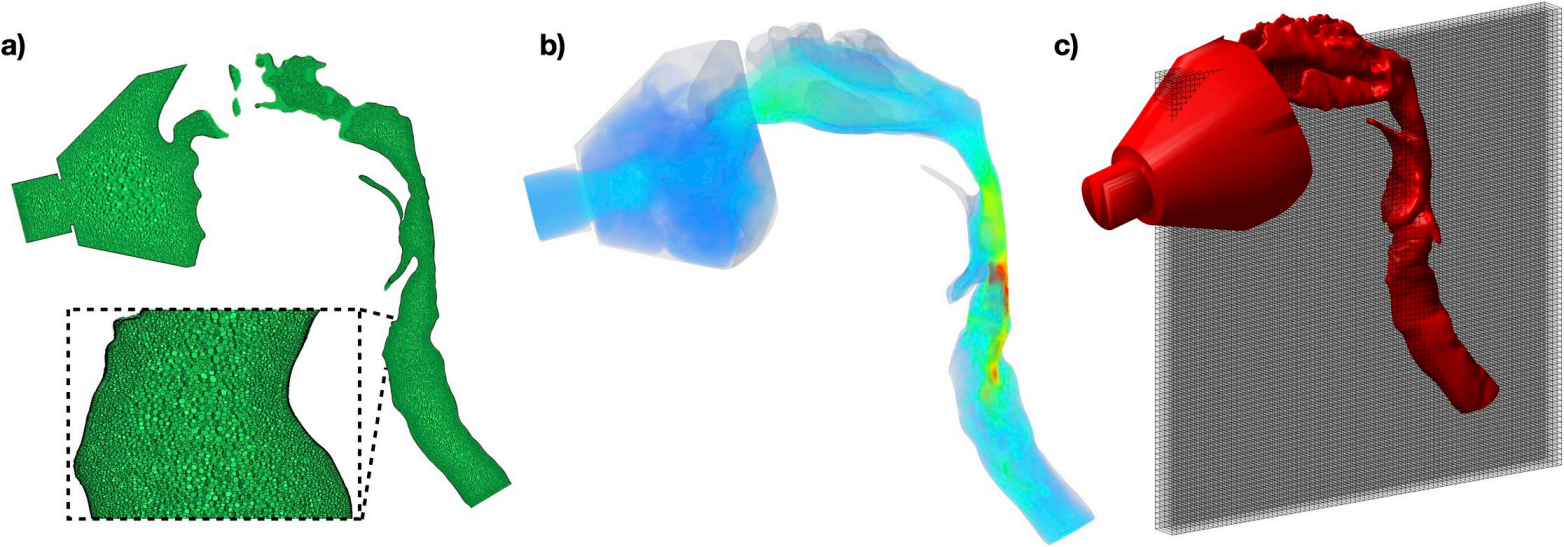
Example of the processing pipeline to facilitate the comparison of CFD and PC MRI data. a). The CFD mesh is generated throughout the 3D airway; cells on a sagittal plane are shown here. b). Representative instantaneous CFD 3D velocity field showing high resolution velocity information throughout the airway. c) 3D airway model derived from ^1^H MRI (red) used to define the 3D virtual surface for CFD, overlaid with cuboids (grey) illustrating the field-of-view and comparatively coarse voxel dimensions of the PC MRI data. These cuboids also illustrate the anisotropic shape of the PC MRI voxels. The mean CFD velocity within each voxel and imaging acquisition period is calculated for comparison to PC MRI.

In order to ensure mesh and temporal discretization independence of the CFD results, the mesh size, temporal discretization, and turbulence modeling approach were all varied in one subject. CFD volume meshes were generated with 10 prism layers at the airway wall and with polyhedral elements in the bulk of the mesh. The height of the first layer was 1x10^-5^ m and the total height of the 10 layers was 4.5x10^-4^; the stretching factor between adjacent layers was 1.3. All mesh generation and flow simulations were performed with Simcenter Star-CCM+ 14.06. For convergence testing, meshes were generated with between 1.7 million and 5.3 million elements and temporal discretizations of 0.1 s to 0.001 s were used. To ensure the finest resolution discretization was suitably well resolved to act as a reference, the size of the mesh was compared to the estimated smallest scale of flow instabilities within the flow, known as the Kolmogorov length scale. This scale was calculated as previously described [16,50]. On average across the mesh, the length scale of mesh elements was 3 times larger than the Kolmogorov length scale. Therefore, although the finest mesh did not resolve all length scales within the flow, the vast majority of flow length scales are resolved and the overall flow results would not be significantly different from the fully resolved simulation, since little kinetic energy is contained within the smallest length scales [13]. As the number of mesh elements was increased from the coarsest resolution towards the finest resolution, the spatially down-sampled velocity field (used later for comparison to PC MRI velocimetry) did not change for meshes above 2.5 million mesh elements. Therefore, this mesh size was used for the other subjects. An image of the mesh at the resolution used in all subjects is shown in Fig 3A. Similarly, as the temporal resolution was decreased from the coarsest value, the CFD velocity results did not change below 0.05 s, therefore this time-step was used for the other subjects. The choice of these parameters produced the same comparison results as that of the higher resolution simulations, but at lower computational cost.

Turbulence modeling can be used to incorporate the effect of flow features smaller than the mesh into the simulation results. Two approaches were compared in this study: unsteady Reynolds-Averaged Navier Stokes (URANS), and large eddy simulation (LES). For the URANS approach, a *k-ω* turbulence model was used [51], and for the LES simulations a wall-adapting local eddy-viscosity model was used [52]. When comparing the velocity results in the foot-to-head direction of these simulations to the PC MRI velocimetry, the results of the LES produced the best correlation (URANS: r = 0.65, LES: r = 0.68), therefore the LES turbulence model was used for all further simulations. An example of an instantaneous 3D velocity field is shown in Fig 3B.

### Dynamic similarity–comparing air and xenon

^129^Xe has a higher density and dynamic viscosity than air and therefore the flow patterns and velocities observed during an inhalation will differ. Since ^129^Xe is not normally inhaled, it is useful to determine what sort of breathing maneuver the ^129^Xe inhalation would represent in air. The concept of dynamic similarity implies that the flow patterns recorded with ^129^Xe will be equivalent to air flowing at a different flow rate. This equivalent flow rate, *Q*_*Air*_, can be calculated by equating the Reynolds numbers of the xenon flow, *Re*_*Xe*_, and the equivalent airflow, *Re*_*Air*_.

$${Re}_{Xe}={Re}_{Air}=\frac{\rho_{Xe}Q_{Xe}D}{\mu_{Xe}A}=\frac{\rho_{Air}Q_{Air}D}{\mu_{Air}A}$$ (1)

where *ρ*_*Xe*_ and *ρ*_*Air*_ are the density of ^129^Xe and air respectively, *μ*_*Xe*_ and *μ*_*Air*_ are the respective dynamic viscosities. *D* is a characteristic dimension of the airway such as the hydraulic diameter, and *A* is the cross-sectional area of the airway. As the geometric parameters, *D* and *A*, do not depend on the gas flowing through the airway, the relationship between the flow rate of ^129^Xe and the equivalent flow rate of air simplifies to:

$$Q_{Air}=Q_{Xe}\frac{\rho_{Xe}}{\mu_{Xe}}\frac{\mu_{Air}}{\rho_{Air}}$$ (2)

Given: *ρ*_*Xe*_ = 5.761 kg.m^-3^; *μ*_*Xe*_ = 2.28 × 10^−5^ Pa.s; *ρ*_*Air*_ = 1.18 kg.m^-3^; *μ*_*Air*_ = 1.81 × 10^−5^ Pa.s; the ratio between *Q*_*Air*_ and *Q*_*Xe*_ is 3.88. This scaling factor can be used to calculate the equivalent velocity of air from the velocity of ^129^Xe. Dynamic similarity also allows the Womersley number for ^129^Xe, *α*_*Xe*_, and air, *α*_*Air*_, to be matched, as follows:

$$\alpha_{Xe}=\alpha_{Air}=D{\left(\frac{\omega_{Xe}\rho_{Xe}}{\mu_{Xe}}\right)}^{\frac{1}{2}}=D{\left(\frac{\omega_{Air}\rho_{Air}}{\mu_{Air}}\right)}^{\frac{1}{2}}$$ (3)

Where *ω*_*Xe*_ and *ω*_*Air*_ are the angular frequencies (i.e. 2π multiplied by the inverse of the duration) of the xenon and air inhalations, respectively. Therefore:

$$\omega_{Air}=\omega_{Xe}\frac{\rho_{Xe}}{\mu_{Xe}}\frac{\mu_{Air}}{\rho_{Air}}$$ (4)

Therefore, while the subjects were asked to perform long, low-velocity inhalations of ^129^Xe, the flow is equivalent to that of shorter, faster breaths of air. For example, an inhalation of ^129^Xe lasting 15 s would equate to $\omega_{Xe}=\frac{2\pi }{15}=0.42$, which is equivalent to *ω*_*Air*_ = 1.62 or an inhalation of air of 3.88 s duration.

The Womersley number for our CFD simulations (Eq 3) is 4.9, based on the values given above and using a characteristic length of 1.5 cm (the hydraulic diameter of the glottis in subject #1). Since this value is greater than 1, transient inertial forces due to the unsteady nature of the breath may be important and this factor led to the methodological choice of a transient simulation approach over computationally less expensive steady-state simulations.

### Comparing velocities from PC MRI and CFD–alignment and resolution

To align the velocity maps produced by CFD and PC MRI for comparison purposes, the high-resolution proton MR images (used to generate the virtual airway surfaces used for the CFD simulations) were registered with the lower-resolution proton MR images taken in the same session as ^129^Xe PC MRI, i.e. with the ^129^Xe coil *in situ* and with the subject in the same position as during the ^129^Xe inhalation. The registration process compares normalized intensities in the high- and low-resolution images and then minimizes the image dissimilarity by moving regions of voxels in one image. The movement of the voxels to provide the lowest image dissimilarity results in a deformation map. To prevent non-physiological changes in the images, a bending energy term is added to the image dissimilarity term, as described by Rueckert et al. [53] and previously described in detail for application to airway CFD models derived from MRI [23]. The resulting deformation field is then applied to the airway surface segmented from the high-resolution proton MR image, to move it into the position in which the PC MRI data was acquired. The result is a high-resolution airway surface in the anatomical position in which images were acquired whilst ^129^Xe was inhaled. All CFD simulations were performed on these airway surfaces.

The velocity field produced by CFD simulations has a much finer spatio-temporal resolution than that derived from PC MRI (~0.1 mm^3^, 0.05 ms vs ~1 mm^2^ in-plane, ~10 mm through-plane, ~3–5 s). Therefore, velocity from all CFD cells that correspond to the volume of each MRI voxel are spatially averaged to produce the mean value for a cell of identical spatial resolution to the PC MRI data. The CFD velocities are averaged both across the PC-MRI slice thickness in the right-left direction, and within the sagittal plane. This process is shown in Fig 3C. The temporal mean over the period of each dynamic PC MR image was also calculated in each cell.

### Statistical analysis

The agreement between velocity maps derived from PC MRI and CFD was assessed by Pearson’s correlation of the velocity values on a voxel-wise basis (statistical significance was assessed at the *P*<0.05 or *P*<0.001 level). Correlation analyses were also performed after down-sampling the PC MRI velocity maps by sliding a 3x3 window across the data and replacing the window with a single averaged velocity pixel value, to evaluate the agreement in coarser spatial regions. Bland-Altman plots of the mean velocity against the difference in velocity between PC MRI and CFD were generated to help identify systematic differences in the two methods. These analyses were performed separately for each directional component of velocity and the velocity magnitude.

## Results

The mean inhalation flow rate of ^129^Xe was ~80 mL/s which by dynamic similarity (described above) is equivalent to a flow rate of air of 310 mL/s, representative of restful breathing [54].

**Fig 4:**
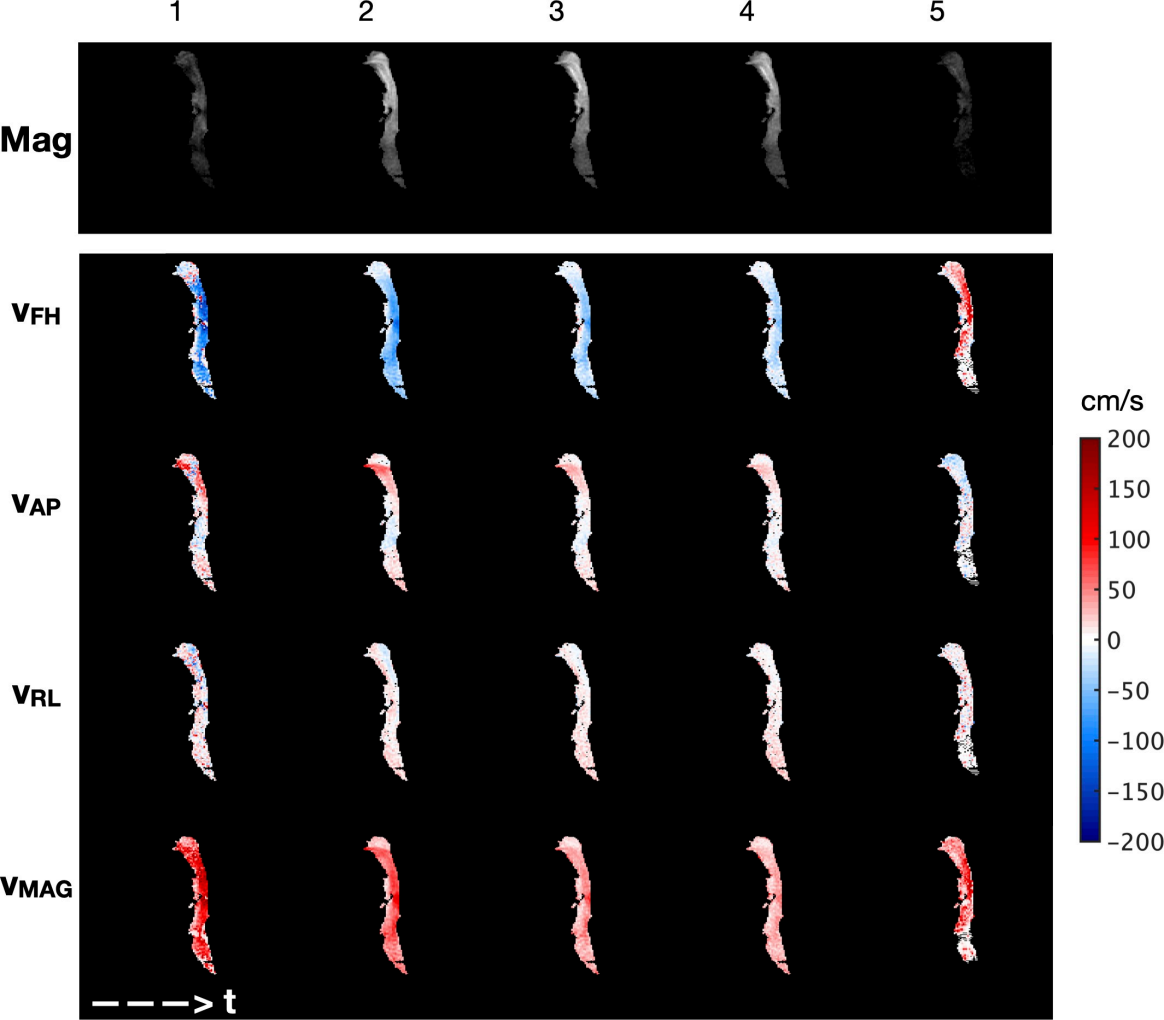
Example ^129^Xe gas PC MRI dataset acquired from subject 1. The first 5 dynamic images from a 10-image time series are shown left-right, where each dynamic scan corresponds to a 3.1 s interval. Top row: MRI magnitude images showing the ^129^Xe gas density in the airway. Lower rows: Foot-head (*v*_*FH*_), anterior-posterior (*v*_*AP*_) and right-left (*v*_*RL*_) components of velocity derived from the dynamic PC MRI time series, and the corresponding velocity magnitude (*v*_*MAG*_, vector sum of the three directional components). Colour key: For *v*_*FH*_, red = flow from foot to head, blue = flow from head to foot; for *v*_*AP*_ red = flow from anterior to posterior, blue = flow from posterior to anterior; and for *v*_*RL*_ red = flow from right to left, blue = flow from left to right. *v*_*MAG*_ is non-directional.

Fig 4 shows the first 5 dynamic images of a 10-image dynamic time-series PC MRI dataset acquired from subject 1, depicting all three components of velocity, in addition to its magnitude. In general, velocity in the foot-to-head direction, *v*_*FH*_, showed the highest velocity values, which is expected as the direction of airflow is largely aligned with the main axis of the airway. Velocity in the anterior-to-posterior direction, *v*_*AP*_, showed moderate-high velocity regions in the nasopharynx and in regions where the airway turns significantly. Few regions of high velocity data were recorded in the right-to-left direction, *v*_*RL*_.

For the dataset in Fig 4, the mean velocity in the foot-head direction (and the mean velocity magnitude) decreased over the first 4 dynamic scans during the slow inhalation period. A reversal in direction of v_FH_ and v_AP_ can be identified in dynamic image #5 indicating that the subject began to exhale, which agrees with the *in*-*situ* flow measurement (note: exhalation data was not considered in subsequent analysis as the composition of the mixture of ^129^Xe and air in the lung prior to inhalation was not known). Beyond dynamic scan #5, the signal-to-noise ratio became insufficient to obtain accurate velocity information, and thus these dynamics were not shown in Fig 4 or considered in further analysis.

**Fig 5:**
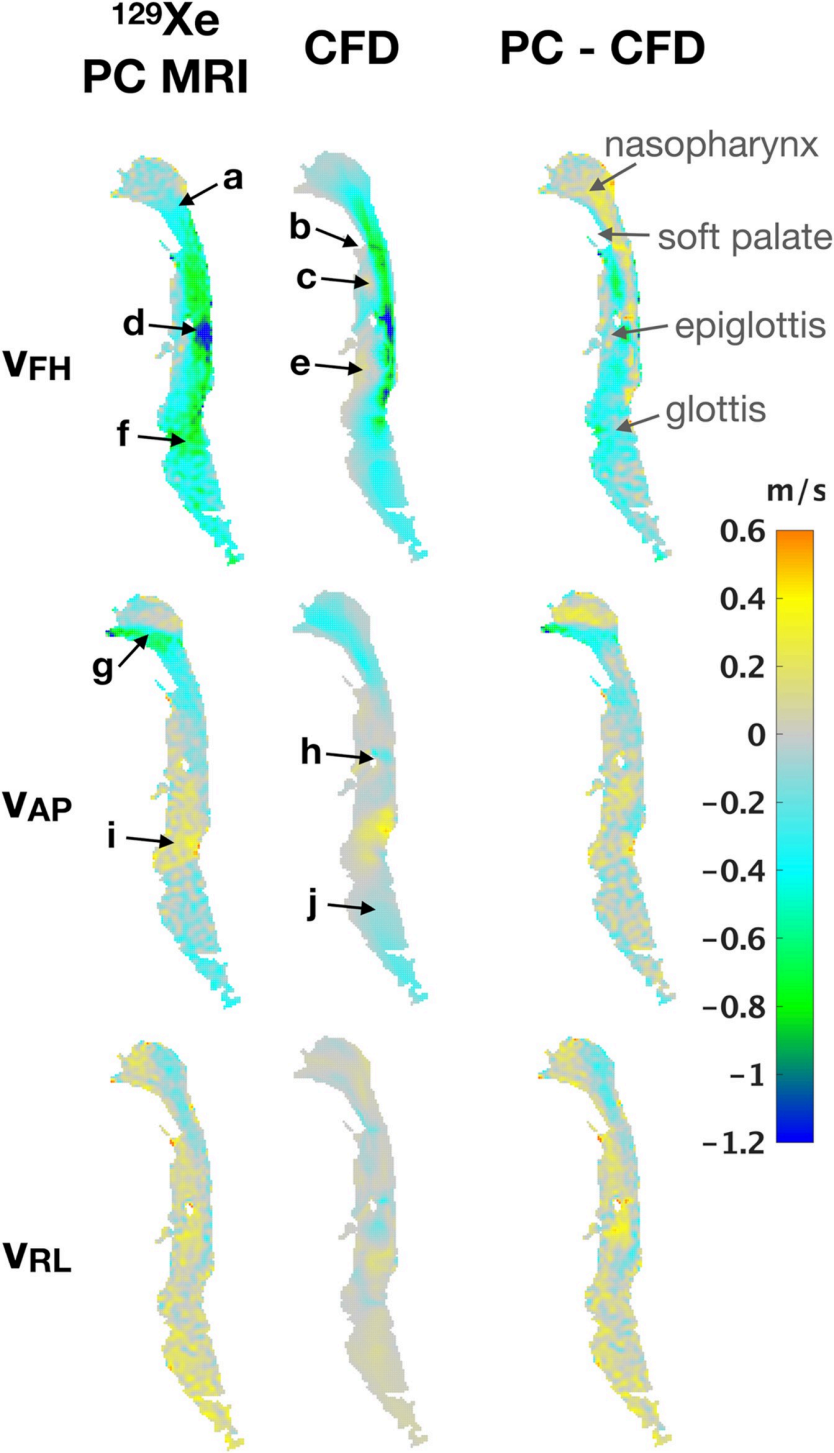
Side-by-side comparison of PC MRI and CFD. Comparison of the foot-to-head, anterior-to-posterior, and right-to-left components of velocity for, left: A ^129^Xe gas PC MRI dynamic image (dynamic image #2 in Fig 4); middle: The CFD “slice” resulting from the regional averaging of the 3D CFD volume in to a 2D slice that corresponds to the PC MRI voxel data, as described in Fig 3); right: Voxel-wise difference between PC MRI and CFD data, highlighting regions of good and poor agreement. Labels a-j highlight important flow features highlighted in the main body text.

Fig 5 shows velocity maps obtained via PC MRI and CFD for the period of the 2^nd^ dynamic image in subject 1. The upper row shows *v*_*FH*_. The velocity maps obtained from the two techniques exhibit many similar flow features; flow accelerates within the nasopharynx (label a) in Fig 5) before separating at the tip of the soft palate b). A low-velocity recirculation region is observed in the anterior oropharynx c), although this region appears larger in the CFD velocity field. As flow passes the tip of the epiglottis, it reaches the highest velocity in this section of airway, resulting in a jet along the posterior wall of the airway d). Again, this jet appears narrower in the CFD velocity map, with a larger low-velocity region anterior to the jet e). Both maps show another high-velocity region where flow passes through the larynx f). *v*_*AP*_ demonstrates similar qualitative agreement. The nasopharynx is dominated by flow in the posterior direction in the lower part of the nasopharynx as the flow turns 90° g). There is a small region of flow in the posterior direction as the flow passes behind the epiglottis h). The flow moves anteriorly to follow the curvature of the airway through the hypopharynx and into the larynx i), before returning to a slightly posterior direction below the glottis j). There is little agreement between the two *v*_*RL*_ maps, attributable to: (i) the general lack of directional airflow and (ii) the fact that uncertainty in PC MRI velocity values increases at smaller absolute velocity values, especially as the maximum velocity encoding is set well above the measured velocities.

**Fig 6:**
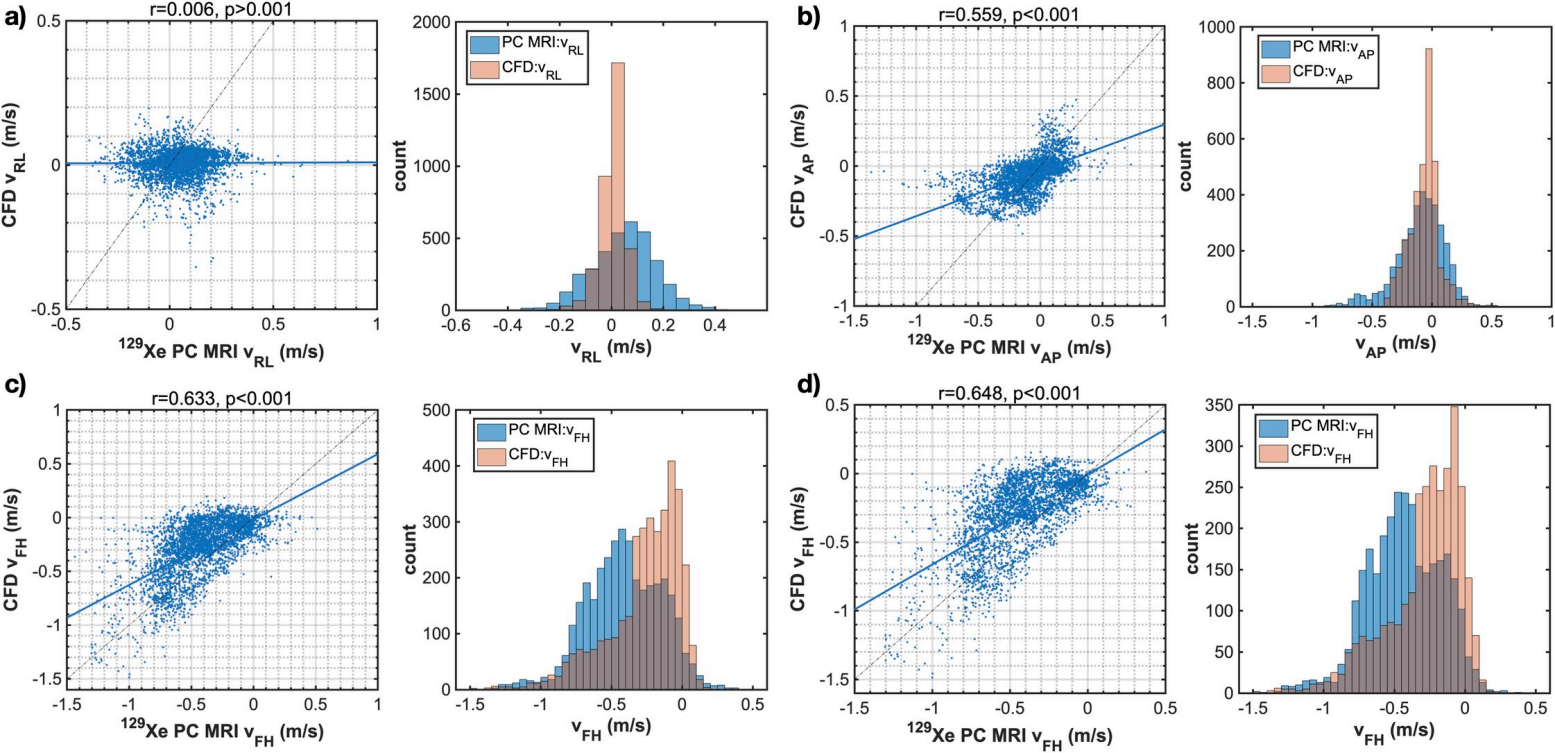
Correlation plots and histograms of agreement for each velocity component: PC MRI and CFD. a) right-left component, b) anterior-posterior component, c) foot-head component. d) data in c) after removal (“erosion”) of the boundary layer of pixels from the 2D maps shown in Fig 5 (Subject 1). Solid blue lines indicate the line of best fit and dashed lines are the identity line. Histograms of velocity values derived from PC MRI and CFD are displayed in blue and red color, respectively.

Quantitative assessment of the agreement between PC MRI- and CFD-derived ^129^Xe gas velocity values in the airways was performed by plotting the correlation and histograms of the voxel-wise velocity values (Fig 6). Pearson’s correlation coefficients were low and statistically insignificant for *v*_*RL*_, whilst coefficients *r* ~ 0.5 for *v*_*AP*_ and > 0.6 for *v*_*FH*_ were observed, both statistically significant at the *P* < 0.001 level. In general, the spread of PC MRI velocity values was greater than that of CFD velocity values, reflected by broader histograms. Comparison between the velocity histograms for *v*_*FH*_ (Fig 6C) reveals more voxels with low velocity in the CFD simulations than in the PC MRI data. This matches the qualitative results above (Fig 5) which show large, slow moving recirculation regions downstream of airway structures such as the epiglottis in the CFD velocity maps. The effect of these regions on the correlation plots is to reduce the slope of the linear line of best fit from the expected value of 1. However, a clear trend of data points lying along a slope of 1 is visible for voxels lying outside of those recirculation regions.

Fig 6D illustrates the effect of eroding the boundary layer of the velocity maps by one pixel, in attempt to remove biases introduced by differing measurement conditions at the boundary (see *Discussion*). In the subsequent analysis, we focus on the dominant flow component, i.e., the foot-head velocity (*v*_*FH*_).

**Fig 7:**
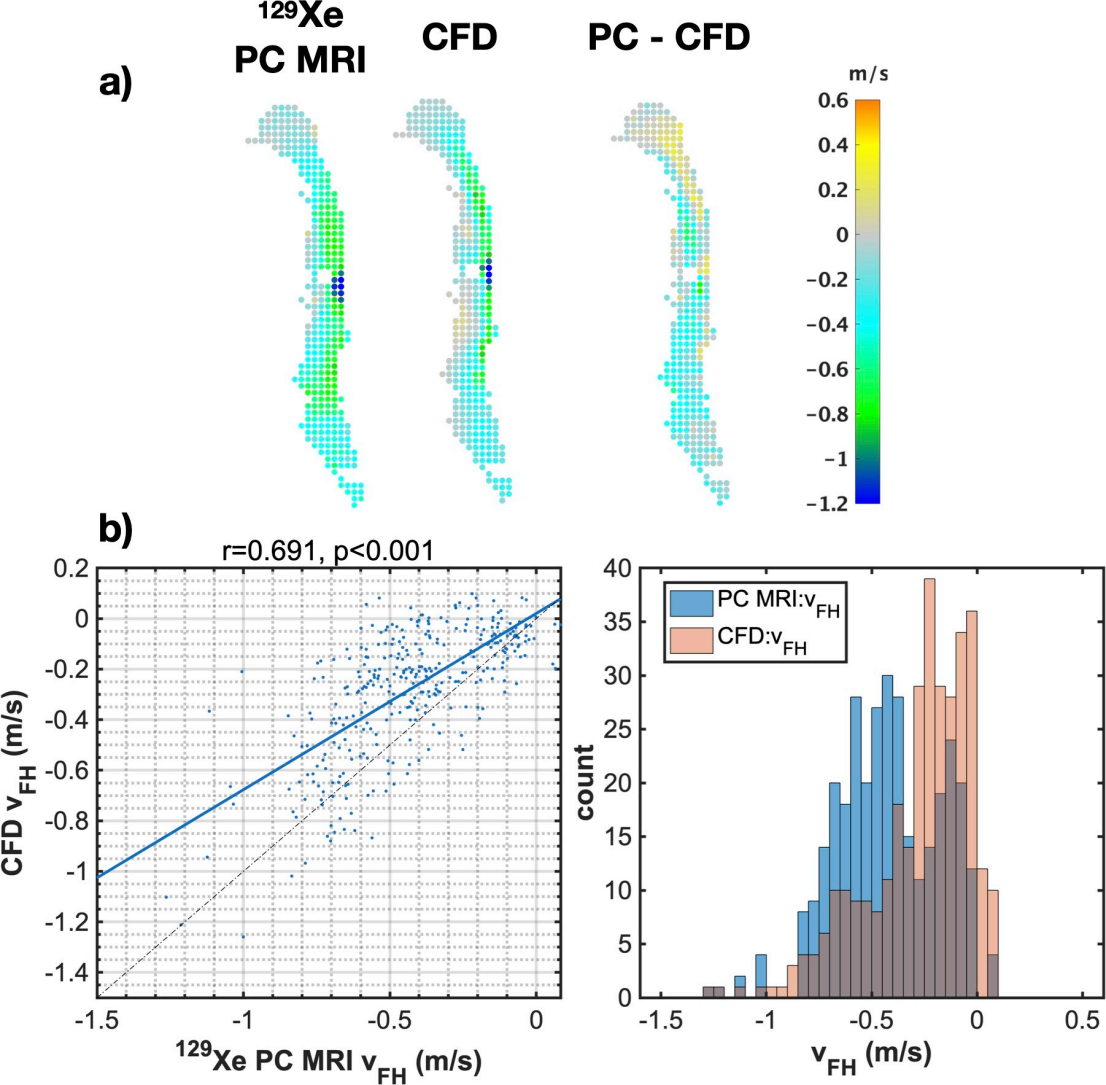
Effects of spatial down-sampling on PC MRI and CFD comparison. a) 2D velocity (*v*_*FH*_) maps for PC MRI and CFD data and the difference between them, and b) correlation plots and histograms presented as in Fig 6, after down-sampling of the PC MRI data in plane by 3x3 voxels and re-sampling the CFD data to the corresponding resolution.

To evaluate the agreement between ^129^Xe gas PC MRI and CFD-derived velocity maps over coarser spatial regions, PC MRI data were down-sampled by applying a 3x3 averaging function to the velocity maps. CFD data were correspondingly re-sampled from the initial high-resolution 3D mesh and the down-sampled 2D spatial maps were compared regionally (Fig 7A) and correlation and histogram plots were generated (Fig 7B). This procedure illustrated an improved agreement in spatial flow patterns and Pearson’s correlation coefficients for *v*_*FH*_.

**Fig 8:**
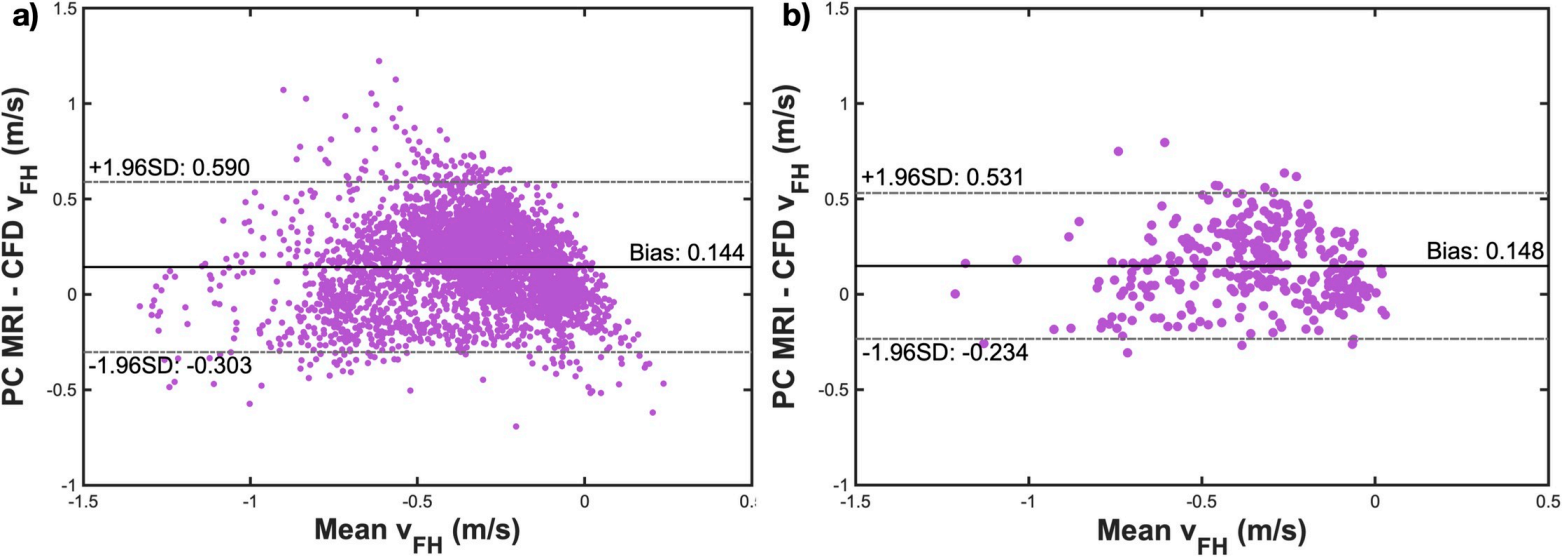
Bland-Altman plots for *v*_*FH*_ between PC MRI and CFD. a) Bland-Altman plot for the voxel-wise mean and difference in *v*_*FH*_ between PC MRI and CFD for subject 1. b) Analogous plot to a) after down-sampling the data in plane by 3x3 voxels.

Systematic differences between ^129^Xe gas PC MRI- and CFD-derived velocity measurements were further assessed by Bland-Altman analysis (Fig 8). Representative Bland-Altman plots of *v*_*FH*_−depicting the mean of PC MRI and CFD *v*_*FH*_ values (x axis) against the absolute difference between the two values (y axis) for each voxel in the image–are shown for the full-resolution and down-sampled velocity distributions. The mean bias across all subjects was low; 0.176 m/s, indicating that CFD velocity values agreed well with, but were slightly lower on average than PC MRI values. The mean lower and upper limits of agreement were -0.534 m/s and 0.886 m/s, respectively across all subjects.

**Fig 9:**
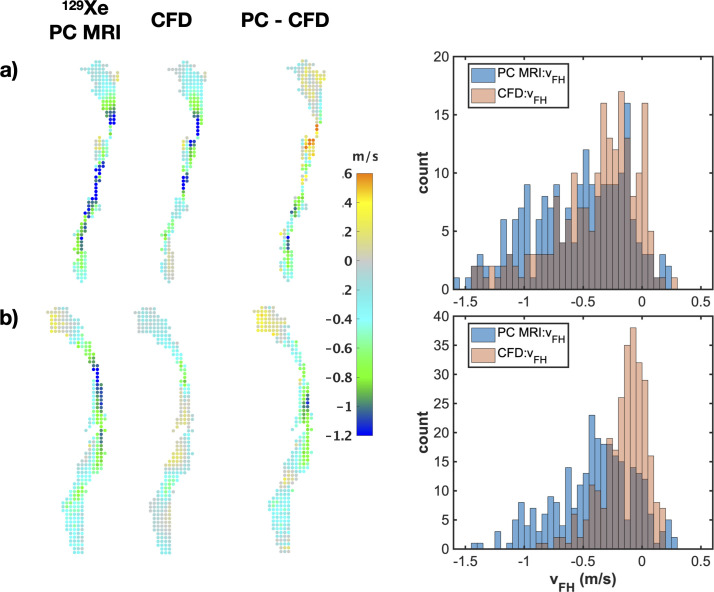
Side-by-side comparison of PC MRI and CFD: Additional examples. Spatial comparison of 3x3 down-sampled foot-head velocity data from PC MRI and CFD, difference maps, and corresponding histograms of velocity values, for a) subject 2 and b) subject 3. Dataset shown is taken from the 3^rd^ temporal dynamic image of the ^129^Xe PC MRI time series.

In Fig 9, representative *v*_*FH*_ data obtained from subjects 2 and 3 is displayed in the form of 3x3-down-sampled velocity maps along with their difference maps and the corresponding velocity histograms. Similar qualitative agreement in gas flow patterns can be observed as with subject 1 (shown previously in Fig 7), with reasonable matching in the spatial location of high and low flow velocities measured at a coarse spatial scale.

## Discussion

CFD simulations of respiratory airflow are widely used to quantify and visualize the function of the human airways. They have previously been validated through comparison to *in vitro* experiments and *in vivo* in rats airways [39–43], but as static experimental models or small animal models do not represent all aspects of human airway physiology, *in vivo* validation is a vital step in achieving clinical adoption of CFD methodology. This study is the first assessment of the similarity between maps of respiratory airflow velocity calculated by CFD simulations and ^129^Xe PC MRI obtained *in vivo* in humans.

To obtain sufficient MRI signal from gas in the airways, hyperpolarized ^129^Xe was inhaled as a high-sensitivity contrast agent. One unavoidable disadvantage of this approach is that ^129^Xe has different aerodynamic properties than air; the density and dynamic viscosity are 4.88 and 1.26 times higher, respectively [55]. Therefore, in order to achieve dynamic similarity (see *Methods*, *Dynamic Similarity–Comparing Air and Xenon)*, subjects were asked to inhale as slowly as possible, with the result that the airflow achieved in the upper airways was representative of inhalation flow rates ~310 mL/s (for air), equivalent to restful breathing. An alternative strategy would be to mix the ^129^Xe with a lighter-than-air gas such as helium to match the density of air. This technique is used in hyperpolarized gas MRI of lung ventilation [56], but such dilution of ^129^Xe would in turn lead to a deleterious reduction in signal-to-noise of PC MRI images assuming the total delivered volume of gas was unchanged.

As anticipated, the highest (and broadest range of) velocity values were measured in the foot-to-head direction, *v*_*FH*_, as the largest component of airflow is aligned with the main axis of the airway. The relatively low flow velocity values and lack of striking flow patterns in the right-left direction (*v*_*RL*_) likely result from the fact that our data represents the contribution from xenon gas flowing across a 10–12.5 mm thick slice in the right-left direction. Thus, we would expect that regions of high and low flow are averaged out over the slice thickness, which is an order of magnitude greater than the in-plane spatial resolution. While the PC MRI slice thickness is relatively coarse, CFD simulations are performed at a much higher spatial resolution and as such, may reveal additional flow features in the right-left direction. The spatial averaging process performed to combine CFD results across the PC MRI slice (see *Comparing Velocities from PC MRI and CFD–Alignment and Resolution*) also has some effect on the velocity profiles in the foot-head and anterior-posterior directions. Qualitatively, we observed that for thin CFD “slices” (~0.1 mm as opposed to 10 mm), the principal flow features observed in the coarser slices were preserved, though the anterior-posterior extent of the jet region at the epiglottic was increased in the fine spatial resolution case. In addition, CFD simulations afford a short *temporal* resolution, whereas we calculated the temporal mean of the CFD results over the acquisition period of each dynamic PC MR image to provide a fair comparison. However, this intrinsically loses information about the dynamics of the flow profile *within* that period. For example, during the acquisition of the first few PC MR images, fine temporal resolution CFD results allow us to track the front of inhaled xenon passing through the airways and visualize the effects of the overall changes in flow-rate during that period.

The correlation coefficient values we report here (*r* ~ 0.6 and above) for the spatial pixel-by-pixel correlation of PC MRI and CFD in *v*_*AP*_ and *v*_*FH*_ are similar, though slightly lower than those reported for *in vitro* experiments with hyperpolarized ^3^He gas MRI [40] and water (^1^H) MRI (*v*_*FH*_ *r*>0.95 for central airway tree, or *r* = 0.86 (*r*^2^ = 0.74) when including upper airways) [41] in airway casts, and *in vivo* hyperpolarized ^3^He MRI in rats (where for *v*_*FH*_ *r* = 0.82 (*r*^2^ = 0.68) over the whole of the airways) [39]. Differences may be attributed to the lower MR signal attainable with ^129^Xe compared to ^3^He and ^1^H, and also the intrinsic advantages of *in vitro* studies or *in vivo* studies in mechanically-ventilated animals; (i) the SNR is typically greater (often resulting from increased scan duration and/or signal averaging), (ii) flow rates can be better controlled.

A further possible cause for differences in PC MRI- and CFD-derived velocity maps reported in this *in vivo* study compared to previous studies is upper airway motion. The CFD geometry was obtained from ^1^H MR images acquired while the subject was breathing restfully, but the ^129^Xe PC MRI data were acquired while the subject was inhaling as slowly as possible. In addition, a different radiofrequency coil was used for MR signal detection due to the different nuclei (^1^H and ^129^Xe MRI) and since these coils were incompatible, the subject may have changed position during the coil switching process. While image registration was performed to account for any bulk subject motion, this may have been imperfect. Subjects may change the position of structures in the upper airway such as the vocal folds and soft palate to widen/narrow the airway in order to control inhalation flow rate. As such, small changes in the position of these structures may explain the observation of smaller high velocity regions in the CFD velocity maps, with larger recirculation regions behind these structures.

Comparing the correlations found between ^129^Xe PC MRI and CFD velocity maps in the airways with previous studies in vascular hemodynamics reveals a similar level of agreement. For example, the r values reported in this study are within the range reported by Isoda et al. [57] Discrepancies between the two methodologies were similar to those reported in this study; e.g. that PC MRI did not reveal recirculation regions that were calculated by CFD [58–60]. Correlation between the two techniques in the airways may differ from that in previously reported vascular studies for several reasons. Respiratory PC MRI involves inhalation of exogeneous ^129^Xe gas as a contrast agent whilst in hemodynamics endogenous blood is imaged directly. In fact, in some cardiovascular studies, PC MRI velocity values are used to *define* the CFD inlet boundary condition [34,57], thus leading to better agreement in the upstream flow rates. In contrast, the present study used the flow of ^129^Xe measured by a flow meter as the inlet flowrate; the accuracy of which is a major limitation of our study. Furthermore, the airway has a highly complex shape, and the narrow and convoluted nasal passages condition the air into the area of interest in this study, which also contains highly dynamic structures such as the tongue, soft palate, and epiglottis.

We have proposed two methods to improve the robustness of our comparisons between PC MRI and CFD; (i) removal of the outer boundary layer of the PC MRI and CFD maps, and (ii) spatial down-sampling of PC MRI and CFD data. At the boundary, the discrepancy between PC MRI and CFD is large, and may be explained by the low velocities of gas at the wall. Fluid near a wall moves much more slowly than fluid in the center of the conduit; this slow-moving region is often termed the “boundary layer”. These low velocity regions will generate less MR signal than high moving flow for several reasons. 1) The partial volume effect, which is a well-known source of error in near-wall MRI flow measurements [61,62]. The finite grid of the MR image is not perfectly conformal with the complex airway anatomy and thus near-wall voxels will comprise tissue, reducing the local signal and potentially skewing the velocity measurements. 2) Inhaled ^129^Xe will take longer to displace the air that was initially inside the airway due to the low velocities in the boundary layer. Therefore, there will be a lower concentration of ^129^Xe in these regions. 3) The slow-moving flow in the boundary layer will also remain in the airway for longer. Hyperpolarized ^129^Xe MR signal decays according to radiofrequency pulse excitation and the longitudinal relaxation time (T_1_), and thus, the longer the ^129^Xe remains in the airway, the more signal decay it will be subjected to. To eliminate this potential source of error, the boundary layer was removed by eroding the mask by 1 voxel at the boundary and the agreement between the two methods was re-evaluated.

Despite our observation of some discrepancies in low velocity voxels between CFD and PC MRI data, it is nevertheless evident from the Bland-Altman analyses (Fig 8) that there are significant discrepancies at all velocities in the range studied. Indeed, even if we exclude data at low velocities the correlation coefficient remains little changed; for *v*_*FH*_ cut-off values between 0 and 0.5 m/s, the correlation coefficient remains ~0.5–0.6. It is unclear as to whether this reflects a true change in the correlation strength or whether the significant reduction in number of datapoints is driving the statistics. There are several reasons why voxels with higher velocity values between the two techniques are mismatched. In particular, the velocity is sensitive to exact geometric position and therefore any image registration errors; such misalignment can affect high flow velocity features in terms of both position and magnitude.

Secondly, in order to evaluate the agreement between PC MRI and CFD flow patterns over more coarsely-defined spatial regions, we spatially down-sampled the results as a means of smoothing out variations in velocity values that may be subject to noise in ^129^Xe PC MRI measurements. Higher resolution spatial comparisons between PC MRI and CFD, while desirable, are limited by the spatio-temporal and SNR encoding efficiency of PC MRI acquisition techniques. Down-sampling provides artificially improved signal-to-noise and thus more robust PC MRI-derived velocity measurements, while still providing regional information (whereas the clinical gold-standard of respiratory airway assessment, spirometry provides no spatial information). In future studies, PC MRI signal could be improved by the use of ^3^He gas, which has a higher gyromagnetic ratio than ^129^Xe but is prohibitively expensive and now difficult to obtain for routine clinical use.

One of the major sources of error in CFD simulations occurs in obtaining the boundary conditions. Firstly, the airway anatomy is obtained via segmentation of proton MRI. Cherobin et al. have shown that varying segmentation approaches can significantly change nasal airflow [63]. While the current study focused on the laryngopharyngeal airway, which is not as narrow as the nasal airways and therefore less sensitive to segmentation errors, velocities will be affected by error in determining the position of the air-tissue boundary. For this study, the largest source of error was associated with the measurement of the inhaled flow rate over time, which was used as the inlet flow condition for the CFD simulations. Errors arose due to difficulties in determining the gas mixture flowing through the flow meter during inhalations for MRI and calibration of the flow meter. While errors in the flow meter calibration may change the magnitude of the flow, the pattern will remain relatively unchanged; previous studies showed that flow patterns are established very quickly and only the magnitude of the velocity maps change significantly through inhalation [16].

The uncertainty in phase contrast MRI velocimetry measurement depends on the SNR and aliasing velocity (venc), and can be estimated using the formula: $\sigma_{v}=\frac{\sqrt{2}}{\pi }\frac{venc}{SNR}$ [64–66]. Taking the second and fifth dynamic images of the PC MRI dataset in Fig 4 to represent examples of good and poor SNR, respectively, we can estimate the SNR as ~24 in a good case and ~7 in a poor case. (SNR was calculated as the ratio of the mean signal within a region of interest placed in the airways at the level of the soft palate, and the standard deviation of the noise in a region of interest placed outside the airways.) Therefore, with an aliasing velocity of 200 cm/s, we can estimate that the uncertainty in velocity is around 4–13 cm/s (i.e. < 10% of venc). This is of the order of the bias between PC MRI- and CFD-derived velocity values and less than half of one standard deviation from the mean difference (see Bland-Altman plots in Fig 8). As such, the uncertainty in PC MRI values may explain some of the discrepancy between PC MRI- and CFD-derived velocity values, though there is a significant fraction of the variance that we hypothesize is accounted for by the other sources of error discussed above. In future works, especially those involving clinical PC MRI velocimetry data, it would be appropriate to set an SNR threshold (for example SNR = 15), below which velocity measurements are not performed due to the associated systematic uncertainty.

Since both CFD velocity maps and PC velocimetry have sources of uncertainty and there is no clinically accepted technique to produce airflow velocity maps, it is not clear whether either of these techniques can be considered “gold standard” measurements. In recent work, Cherobin et al. used rhinomanometry–a clinical technique to measure nasal pressure and flow–as the “gold-standard” with which to compare CFD measurements of nasal resistance [67]. They reported a weak correlation between CFD measurements and rhinomanometry *in vivo* (*r*~0.4), and that CFD tended to predict lower values of nasal resistance, attributing this to intrinsic variability in rhinomanometry (~ 15%). On the other hand, to confirm the reliability of nasal CFD simulations, CFD and *in vitro* rhinomanometry were compared, which yielded an excellent correlation (*r*~0.96). It is therefore challenging to conclude that one of these measurements should be used as the gold standard with which to compare the other, and we can conclude that both measurements may be valid but fundamental differences in the technique lead to systematic biases and poor *in vivo* correlation. As mentioned above, CFD has been used previously as the gold standard with which to compare PC MRI, i.e. in exactly the opposite manner to the present work [57]. While these various techniques demonstrate differences in their results, in the absence of an accepted “gold standard” for regionally mapping airflow in the upper airway, it is difficult to define what level of agreement between approaches constitutes validation. Nevertheless, without further data, it is premature to term upper airway PC MRI a “gold-standard”, as is now the case for cardiac hemodynamics PC MRI [28]. Therefore, the goal of the present paper was to demonstrate the broad agreement between CFD and PC MRI, and to illustrate the sources of error which may lead to discrepancies in the results.

This study is the first *in vivo* human comparison of CFD simulations of respiratory airflow and establishes a method for future validation of CFD simulation results by comparison to PC MRI velocity maps. Agreement was similar to that found in vascular hemodynamics studies, although somewhat lower than in previous *in vitro* airway studies, as expected due to constraints in acquiring sufficient signal (SNR ≳ 20) [39] at high resolution (~1–2 mm in plane) during a short and irregular human inhalation. Qualitative agreement between the two techniques was good, with high and low velocity regions and features such as high flow jets occurring in the same spatial locations. This study lays the groundwork for future validation studies in healthy subjects and in patients with airway disease. Such future validation studies based on the techniques demonstrated here will aid the clinical adoption of CFD simulations as a means to assess airway disease and treatment strategies.

## Supporting information

S1 Data (ZIP)
